# Rapid diagnosis of SARS-CoV-2 pneumonia on lower respiratory tract specimens

**DOI:** 10.1186/s12879-021-06591-w

**Published:** 2021-09-08

**Authors:** Vanessa De Pace, Patrizia Caligiuri, Valentina Ricucci, Nicola Nigro, Barbara Galano, Valeria Visconti, Giorgio Da Rin, Bianca Bruzzone

**Affiliations:** 1 Hygiene Unit, San Martino Policlinico Hospital, IRCCS for Oncology and Neurosciences, Genoa, Italy; 2 Laboratory Medicine, San Martino Policlinico Hospital, IRCCS for Oncology and Neurosciences, Genoa, Italy

**Keywords:** Rapid PCR, Bronchoalveolar lavage (BAL), Bronchoaspirates (BAS), SARS-CoV-2

## Abstract

**Background:**

The ongoing SARS-CoV-2 pandemic requires the availability of accurate and rapid diagnostic tests, especially in such clinical settings as emergency and intensive care units. The objective of this study was to evaluate the diagnostic performance of the Vivalytic SARS-CoV-2 rapid PCR kit in lower respiratory tract (LRT) specimens.

**Methods:**

Consecutive LRT specimens (bronchoalveolar lavage and bronchoaspirates) were collected from Intensive Care Units of San Martino Hospital (Genoa, Italy) between November 2020 and January 2021. All samples underwent RT-PCR testing by means of the Allplex™ SARS-CoV-2 assay (Seegene Inc., South Korea). On the basis of RT-PCR results, specimens were categorized as negative, positive with high viral load [cycle threshold (Ct) ≤ 30] and positive with low viral load (Ct of 31–35). A 1:1:1 ratio was used to achieve a sample size of 75. All specimens were subsequently tested by means of the Vivalytic SARS-CoV-2 rapid PCR assay (Bosch Healthcare Solutions GmbH, Germany). The diagnostic performance of this assay was assessed against RT-PCR through the calculation of accuracy, Cohen’s *κ*, sensitivity, specificity and expected positive (PPV) and negative (NPV) predictive values.

**Results:**

The overall diagnostic accuracy of the Vivalytic SARS-CoV-2 was 97.3% (95% CI: 90.9–99.3%), with an excellent Cohen’s* κ* of 0.94 (95% CI: 0.72–1). Sensitivity and specificity were 96% (95% CI: 86.5–98.9%) and 100% (95% CI: 86.7–100%), respectively. In samples with high viral loads, sensitivity was 100% (Table 1). The distributions of E gene Ct values were similar (Wilcoxon’s test: *p* = 0.070), with medians of 35 (IQR: 25–36) and 35 (IQR: 25–35) on Vivalytic and RT-PCR, respectively (Fig. 1). NPV and PPV was 92.6% and 100%, respectively.

**Table 1:** Demographic characteristics and data sample type of the study cases (N = 75)

Male, N (%)	56 (74.6%)
Age (yr), Median (IQR)	65 (31–81)
BAS, N (%)	43 (57.3%)
Negative	30.2%
Positive—High viral load [Ct ≤ 30]	27.9%
Positive—Low viral load [Ct 31–35]	41.9%
BAL, N (%)	32 (42.7%)
Negative	37.5%
Positive—High viral load [Ct ≤ 30]	40.6%
Positive—Low viral load [Ct 31–35]	21.9%

Data were expressed as proportions for categorical variables. Specimens were categorized into negative, positive with high viral load [cycle threshold (Ct) ≤ 30] and positive with low viral load (Ct of 31–35). *BAS* bronchoaspirates, *BAL* bronchoalveolar lavage, Ct cycle threshold

**Fig. 1:**
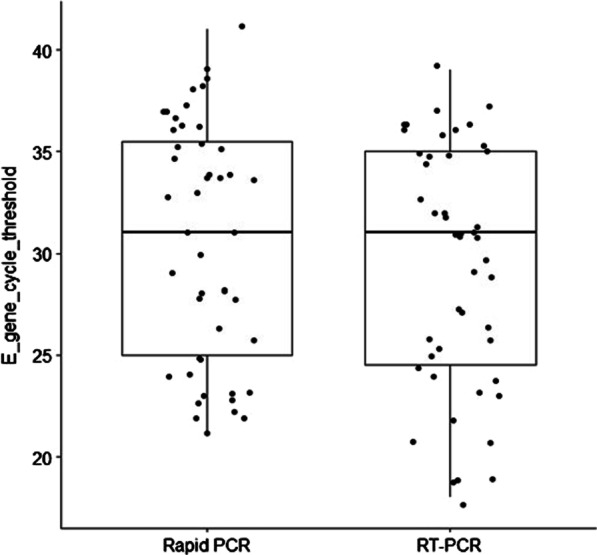
Distribution of E gene cycle threshold values of the rapid PCR and RT-PCR

**Conclusions:**

Vivalytic SARS-CoV-2 can be used effectively on LRT specimens following sample liquefaction. It is a feasible and highly accurate molecular procedure, especially in samples with high viral loads. This assay yields results in about 40 min, and may therefore accelerate clinical decision-making in urgent/emergency situations.

## Background

Global research is committed to exploring every aspect of the coronavirus disease-2019 (COVID-19) outbreak and restoring the normal level of public health. The rapid identification and monitoring of COVID-19 patients is cited as an important strategy in emergency and intensive care units (ICU) [1, 2].

Molecular diagnostic assays, in combination with chest radiography, continue to be gold standard tests for the laboratory diagnosis of the severe acute respiratory syndrome coronavirus 2 (SARS-CoV-2) pneumonia [3].

The US Centers for Disease Control and Prevention (CDC) recommend taking nasopharyngeal swabs (NPS) as the first-choice method of collecting samples for real-time reverse transcriptase polymerase chain reaction (RT-PCR) [4]. During symptom onset in SARS-CoV-2 patients, high viral nucleic acid shedding patterns have been detected in upper-respiratory-tract (URT) specimens, NPS or oropharyngeal swabs (OPS) [5]. However, the critical conditions of patients with severe acute respiratory infection (SARI) who undergo mechanical ventilation do not usually allow the collection of URT specimens. Furthermore, some cases of SARI due to SARS-CoV-2 may present typical chest images, anosmia or ageusia, even if the NPS is RT-PCR negative. [6, 7]. To improve the detection rate and reduce the false-negative rate in these cases, lower respiratory tract (LRT) specimens (i.e. bronchoalveolar lavage, BAL; bronchoaspirates, BAS; sputum; tracheal aspirate) should be used for testing in highly suspect patients [7]. SARS-CoV-2 displays active replication in pulmonary sites, as is revealed by the higher excretion kinetics in sputum than in URT samples [8].

In COVID-19 patients undergoing mask ventilation or automated device ventilation in the emergency setting, highly accurate and sensitive rapid tests would help to establish the laboratory diagnosis and clinical prognosis quickly and efficiently.

In this study, we compared the performance of the Vivalytic SARS-CoV-2 molecular platform (Bosch Healthcare Solutions GmbH; Stuttgarter, Germany) with that of the RT-PCR assay.

## Methods

A sample of consecutive LRT specimens (BAL and BAS) were collected from COVID-19 symptomatic patients in the ICUs at San Martino Hospital (Genoa, Italy) between November 2020 and January 2021. A 1:1:1 sampling ratio was used in order to achieve a sample size of 75. Following collection and transport, each sample was analyzed for the qualitative detection of SARS-CoV-2 RNA by means of the Allplex™ 2019-nCoV assay (Seegene Inc.; Seoul, South Korea), according to the manufacturer’s instructions. RT-PCR for detection of the E, RdRp/S and N genes was performed in a Bio-Rad CFX96 Deep Well real-time PCR detection system (Bio-Rad, Hercules; California, USA), after viral RNA extraction by means of an NX-48 viral nucleic acid extraction kit (Genolution, Seoul, South Korea). On the basis of RT-PCR results, specimens were categorized as negative, positive with high viral load [cycle threshold (Ct) ≤ 30] and positive with low viral load (Ct of 31–35). The Vivalytic SARS-CoV-2 test was subsequently performed on each specimen.

Vivalytic SARS-CoV-2 is a portable device for molecular diagnostics, and is able to perform the most common PCR procedures fully automatically. A sample is loaded into a specific cartridge after a manual, pre-analytical step that includes both sample preparation and addition.

The Vivalytic SARS-CoV-2 analyzer requires the samples to be collected in a guanidine thiocyanate-based medium eNAT® (COPAN Diagnostics Inc.; Murrieta, USA), which stabilizes the viral RNA and completely inactivates the microbial viability. To dissolve the mucous respiratory specimens, the sample is pre-treated before inoculation into the test cartridge; BAL and BAS specimens were pre-treated with sputasol (Sputasol Liquid, Oxoid Limited; Basingstoke, UK) in a 1:1 ratio (500 µl of sample and 500 µl of SL), vortexed and, after 10 min added to 1 ml of eNAT®.

This singleplex test, which is based on the detection of the E gene, is performed on a specimen volume of 300 µl in 39 min.

In order to assess the test specificity for SARS-CoV-2, the Vivalytic test was simultaneously performed on LRT samples positive for other respiratory viruses (Coronavirus OC43, Rhinovirus type A, Metapneumovirus, Parainfluenza virus type 3, Influenza virus A; Respiratory Syncytial Viruses type B) detected in the 2019/2020 influenza season and stored at − 80 °C. Allplex™ Respiratory Panel Assays (Seegene Inc.; Seoul, South Korea) were used to identify these agents of respiratory infection. The respiratory viruses used to evaluate the specificity of the SARS-CoV-2 Vivalytic were selected on the basis of the viruses detected in LTR samples during the 2019/2020 influenza season.

Data were collected on demography and clinical characteristics: i.e. sex, age, comorbidities, time of illness onset, the radiological appearance of viral pneumoniae, COVID-19-related symptoms and death. Symptoms were defined as common (fever higher than 37.8 °C, troublesome dry cough, fatigue), uncommon (diarrhea, headache, muscle pain, nausea, vomiting, chills, dizziness, loss of sense of smell and taste) and severe (breathless on light exertion, chest pain, loss of speech and movement) according to World Health Organization Guidelines for COVID-19.

Data were expressed as medians with inter-quartile ranges (IQRs) for continuous variables and as proportions for categorical variables. The diagnostic performance of the rapid PCR in comparison with RT-PCR was assessed by calculating accuracy, Cohen’s κ, sensitivity, specificity and expected positive (PPV) and negative (NPV) predictive values. Non-parametric Wilcoxon and Mann–Whitney tests were used to compare dependent and independent continuous variables, respectively.

The Open Source Epidemiologic Statistics for Public Health (OpenEpi, https://www.openepi.com/) and GraphPad Prism (8.0 version) was used for analyses. A *p* value < 0.05 was considered statistically significant.

## Results

### Clinical LRT specimens

The main demographic characteristics of the patients, i.e. sex and age, and the biological features of the study specimens, i.e. BAL or BAS, are shown in Table 1. The samples were predominantly from males (74.6%) and the overall median patient age was 65 (IQR: 58–72.2) years without major differences among groups: 0 (72 years, IQR = 58.5–76.5), 1 (61 years, IQR = 58–69) and 2 (63 years, IQR = 55–68.5). Samples with negative, low and high viral loads were equally distributed (*z*-test: *p* = 0.21) between the types of specimens: BAS (57.3%) and BAL (42.7%).

The main pre-existing comorbidities observed in the study patients were hypertension (N = 18, 24%), cardiovascular disease (N = 9, 12%) and diabetes (N = 8, 10.6%). Obesity, benign prostatic hypertrophy, chronic renal failure, chronic liver failure, cancer, previous tuberculosis, major depressive disorder, thyroid disease, and dysmetabolic disorders were noted as minor comorbidities, with an overall prevalence of < 10%. Less than 14% of the study patients did not suffer from any previous disorders; these cases were highly predominant in groups 0 and 2.

Common, uncommon and severe symptoms were noted in 90% (N = 45), 12% (N = 6) and 48% (N = 24) of the study population, respectively. The most frequent COVID-19-related symptoms observed were fever (N = 33, 66%), troublesome dry cough (N = 11, 22%) and dyspnea (N = 26, 52%).

In our analysis of clinical data, we calculated the median times and IQR, in days, from the onset of symptoms to SARS-CoV-2 testing in group 1 (19.5 days, IQR = 9.7–24.7) and group 2 (39 days, IQR = 29–95). Patients with low viral load displayed a longer median time from symptom onset to sample testing (19.5 vs 39 days, Mann–Whitney *U*-test *p* < 0.001), as expected. However, the overall median times between illness onset and the presence of typical ground-glass opacity in the lung on screening chest X-rays was 6 days (IQR = 3–7.5) without any difference between group 1 (6 days, IQR = 3.2–8.5) and 2 (6 days, IQR = 3–7.5), as expected in the common clinical course of COVID-19 patients [9].

Comorbidities data were collected for group 0, 1 and 2, while the time from illness onset to sample collection and to the radiological appearance of viral pneumonia and COVID-19-related symptoms were analyzed only in groups 1 and 2, as group 0 included negative specimens. However, it is important to report that 7/25 samples from group 0 were collected from patients with previous SARS-CoV-2 infection without any history of COVID-19 re-infection.

Comorbidities and symptoms data, the time from illness onset to specimen collection and to the typical viral pneumonia on screening chest X-rays were reported in Table 2.

**Table 2:** Clinical characteristics of the study patients

Comorbidities (N = 75), N (%)	65 (86.6%)
Hypertension	18 (24%)
Cardiovascular disease	9 (12%)
Diabetes	8 (10.6%)
COVID-19-related symptoms (N = 50), N (%)	
Common	45 (90%)
Uncommon	6 (12%)
Severe	24 (48%)
From symptoms onset to SARS-CoV-2 testing (N = 50), Median (IQR)	
Group 1	19.5 days (9.7–24.7)
Group 2	39 days (29–95)
From illness onset to viral pneumonia on chest X-rays (N = 50), Median (IQR)	
Group 1 and 2	6 days (3–7.5)

Data were expressed as medians with inter-quartile ranges (IQRs) for continuous variables and as proportions for categorical variables. Comorbidities data were collected for group 0, 1 and 2, while the time from illness onset to sample collection and to the radiological appearance of viral pneumonia and COVID-19-related symptoms were analyzed only in groups 1 and 2, as group 0 included negative specimens. Symptoms were defined as common (fever higher than 37.8 °C, troublesome dry cough, fatigue), uncommon (diarrhea, headache, muscle pain, nausea, vomiting, chills, dizziness, loss of sense of smell and taste) and severe (breathless on light exertion, chest pain, loss of speech and movement) according to World Health Organization Guidelines for COVID-19

Furthermore, only 8% (4/50) of SARS-CoV-2 infections in group 1 and 2 were nosocomial cases, i.e. COVID-19 occurred through healthcare transmission during the hospital stay.

Finally, death was recorded in 36%, 56% and 32% of patients in groups 0, 1 and 2, respectively. Excluding the cases with previous COVID-19 infection in group 0, the incidence of death was 33%.

### Accuracy, sensitivity and specificity of rapid PCR

The overall diagnostic accuracy of the rapid PCR was 97.3% (95% CI: 90.9–99.3%), with an excellent Cohen’s k of 0.94 (95% CI: 0.72–1). Sensitivity and specificity were 96.0% (95% CI: 86.5–98.9%) and 100% (95% CI: 86.7–100%), respectively. Indeed, only two false negative results were found, both in BAL samples with low viral loads. Therefore, in samples with high viral loads, sensitivity was 100% (Table 3). NPV and PPV was 92.6% and 100%, respectively.

**Table 3:** Sensitivity and specificity of the rapid PCR assay

Category	Sensitivity, *%*	Specificity, *%*
Negative (N = 25)	NA	100
High viral load (N = 25)	100	NA
Low viral load (N = 25)	92.0	NA
Total (N = 75)	96.0	100

The distributions of E gene Ct values were similar on rapid PCR and RT-PCR (Wilcoxon’s test: *p* = 0.070), with medians of 35 (IQR: 25–36) and 35 (IQR: 25–35), respectively (Fig. 1).

The Vivalytic SARS-CoV-2 assay showed no cross-reactivity on testing samples positive for other respiratory viruses, including other coronaviruses.

## Discussion

In this study, the Vivalytic assay proved to be useful tool as a single molecular testing system for the detection of SARS-CoV-2 in LRT specimens. In particular, in comparison with the gold standard RT-PCR, this rapid PCR assay reached a satisfactory level of accuracy. To the best of our knowledge, our study is the first to evaluate the diagnostic performance of this molecular platform in a large number of LRT specimens. Indeed, Vivalytic SARS-CoV-2 has previously been evaluated, together with the Cepheid Xpert® Xpress SARS-CoV-2 assay (Cepheid Inc., Sunnyvale, U.S.A.) as a cartridge-based nucleic acid amplification testing system on various respiratory specimens, though only on a small sample [10]. In that study, only six positive LRT samples and one negative sample were processed to test the diagnostic features of this rapid molecular test, and low values of sensitivity were reported; moreover, phosphate buffered saline was used as a buffer, instead of the correct eNAT®, as declared [10].

In our study, we also recorded the main clinical data from our subjects. High levels of comorbidities were registered—a clinical finding that is associated with a higher risk of severe COVID-19 infection and adverse outcomes, as reported in other studies [11, 12]. As expected, the median time from illness onset to sample collection was longer in group 2 patients, who had a low viral load, than in group 1 patients, who had high viral loads. However, in group 1, the median time from symptom onset to sample collection was more than 14 days, a time interval that was associated with 100% positivity in LRT specimens in a recent study [13]. Up to about one month after the onset of illness, our samples with low and high viral loads were confirmed as positive by the rapid molecular test, suggesting an optimal diagnostic performance.

Our clinical findings should be interpreted in light of an important limit. Study patients were only hospitalized in ICUs, and were therefore in severe clinical conditions. The incidence of death was higher in group 1 than in groups 0 and 2. This observation is in line with the fact that more group 1 patients had comorbidities (92% versus 80% in group 2 and 88% in group 0). Thus, COVID-19 infection might not have been the main cause of death, but rather the final event which proved fatal in patients with severe disabling diseases.

Various clinical conditions may make it difficult to obtain an NPS, which is the most commonly processed sample type for the diagnosis of COVID-19; other biological materials may therefore need to be analyzed in order to exclude SARS-CoV-2 pneumonia. This situation is particularly relevant if the NPS sample is negative but some the patient’s clinical conditions are suggestive of SARS-CoV-2 infection. The BAL and BAS collection procedures have the disadvantage of being invasive. However, the PCR of LRT specimens may be more sensitive than that of NP swabs [14]. Indeed, some case reports have documented discrepant PCR results between the two types of samples, with LRT samples being positive and URT samples being negative in the same patient [7, 15].

Recently, a case of SARS-CoV-2 transmission during lung transplantation was reported, even though the donor had tested negative on pre-implantation NPS. The assessment of LRT specimens from potential lung donors should therefore be preferred for virological screening prior to transplantation [16]. Similar investigations should also be recommended in the case of other organ transplants, as SARS-CoV-2 has displayed wide tropism in many types of sample (plasma, rectal swabs, stool, urine, kidney and lung tissues) [17].

Analogously, in the critical conditions of respiratory failure (i.e., when patients are intubated), the collection of LRT material is a valid strategy [18]. Indeed, in such circumstances, it is of crucial importance to collect the right type of specimen from a given patient, in order to reduce the incidence of false-negative PCR test results [18].

As Vivalytic COVID-19 is a diagnostic system intended for use on one sample at time, it cannot meet the needs of a high-workflow COVID-19 laboratory. Indeed, it is important to underline that this procedure may be useful to meet low-volume requests for rapid diagnosis, e.g. for SARI patients with pneumonia of unknown etiology and negative results of NPS for SARS-CoV-2, in the pre-operative evaluation of trauma patients or of those undergoing transplantation, particularly of the lung.

We noted two main study limitations. First, we tested only BAL and BAS samples, our results may be not generalizable to other lower respiratory materials, such as tracheal aspirates and sputum. However, since these samples have similar viscosity to that of BAS and BAL material, we believe that the same pre-treatment protocol used in this study may be applied to these specimens. Second, the sample size of this study was limited and skewed towards positive samples. This latter phenomenon may be explained by the time period of the study, which was conducted at the height of the second pandemic wave. On the other hand, the diagnostic accuracy of Vivalytic SARS-CoV-2 on LRT specimens was in line with that declared by the manufacturer for NP samples. Therefore, we believe that this limitation had little impact on our results.

## Conclusions

We reported the results on the diagnostic evaluation of the Vivalytic SARS-CoV-2 rapid PCR assay on LRT specimens. This molecular platform is simple, rapid and fully automated, and requires little pre-treatment of materials and little training time. It may therefore help to accelerate the diagnosis, therapeutic decision-making and prognosis of patients with urgent/emergency conditions, thereby optimizing the management of the ICU [2].

## Data Availability

The datasets used and/or analysed during the current study are available from the corresponding author on reasonable request.

## Abbreviations

COVID-19: Coronavirus disease-2019
SARS-CoV-2: Severe acute respiratory syndrome coronavirus-2
ICU: Intensive care units
CDC: Centers for Disease Control and Prevention
NPS: Nasopharyngeal swab
RT-PCR: Real-time reverse transcriptase polymerase chain reaction
SARI: Severe acute respiratory syndrome
LRT: Lower respiratory tract
BAL: Bronchoalveolar lavage
BAS: Bronchoaspirates
